# Transmembrane Collagen XVII Modulates Integrin Dependent Keratinocyte Migration via PI3K/Rac1 Signaling

**DOI:** 10.1371/journal.pone.0087263

**Published:** 2014-02-05

**Authors:** Stefanie Löffek, Tiina Hurskainen, Joanna Jackow, Florian Christoph Sigloch, Oliver Schilling, Kaisa Tasanen, Leena Bruckner-Tuderman, Claus-Werner Franzke

**Affiliations:** 1 Department of Dermatology and Venerology, University Medical Center Freiburg, Freiburg, Germany; 2 Department of Dermatology, Oulu Center for Cell-Matrix Research, University of Oulu, and Clinical Research Center, Oulu University Hospital, Oulu, Finland; 3 Institute of Molecular Medicine and Cell Research, University of Freiburg, Freiburg, Germany; 4 Faculty of Biology, University of Freiburg, Freiburg, Germany; 5 Bioss Centre for Biological Studies, University of Freiburg, Freiburg, Germany; 6 Freiburg Institute of Advanced Studies, School of Life Sciences – LifeNet, University of Freiburg, Freiburg, Germany; University of Bergen, Norway

## Abstract

The hemidesmosomal transmembrane component collagen XVII (ColXVII) plays an important role in the anchorage of the epidermis to the underlying basement membrane. However, this adhesion protein seems to be also involved in the regulation of keratinocyte migration, since its expression in these cells is strongly elevated during reepithelialization of acute wounds and in the invasive front of squamous cell carcinoma, while its absence in ColXVII-deficient keratinocytes leads to altered cell motility. Using a genetic model of murine *Col17a1^−^* ^***/****−*^ keratinocytes we elucidated ColXVII mediated signaling pathways in cell adhesion and migration. *Col17a1^−^* ^***/****−*^ keratinocytes exhibited increased spreading on laminin 332 and accelerated, but less directed cell motility. These effects were accompanied by increased expression of the integrin subunits β4 and β1. The migratory phenotype, as evidenced by formation of multiple unstable lamellipodia, was associated with enhanced phosphoinositide 3-kinase (PI3K) activity. Dissection of the signaling pathway uncovered enhanced phosphorylation of the β4 integrin subunit and the focal adhesion kinase (FAK) as activators of PI3K. This resulted in elevated Rac1 activity as a downstream consequence. These results provide mechanistic evidence that ColXVII coordinates keratinocyte adhesion and directed motility by interfering integrin dependent PI3K activation and by stabilizing lamellipodia at the leading edge of reepithelializing wounds and in invasive squamous cell carcinoma.

## Introduction

Classical type I hemidesmosomes (HDs) are cell-matrix junctions that provide tissue integrity by anchoring epithelial cells to the basement membrane. They contain a number of interacting components: the transmembrane proteins collagen XVII (ColXVII) and α6β4 integrin, which bind to laminin 332 (LN332) in the basement membrane, and the intracellular linker proteins bullous pemphigoid antigen 230 (BP230) and plectin, which bind to the intermediate filament cytoskeleton. Mutations in the genes encoding HD proteins are associated with hereditary human diseases of the epidermolysis bullosa group that manifest with chronic skin fragility and blistering [1].

Disassembly of HDs is required during biological and pathological processes such as tissue repair, tumor cell migration and invasion. These processes are characterized by a balanced combination of cell-matrix attachment and detachment, implicating that HD components are also involved in the regulation of cell motility [2], [3]. Cell migration involves an initial formation of protrusions at the leading edge (lamellipodia) with actin-rich membrane ruffles, followed by their attachment to the extracellular matrix and the formation of focal adhesions at the front and, finally, concomitant detachment of adhesive contacts at the rear of the cell. Efficient migration requires an optimum of adhesion strength; too weak adhesion is inadequate for cell traction, whereas too strong adhesion is incompatible with migration [4], [5].

The role of ColXVII in cell adhesion and migration is supported by genetic evidence derived from junctional epidermolysis bullosa (JEB), a disease with skin fragility and mechanically induced skin blistering. *In vitro* studies with primary JEB keratinocytes demonstrated that low abundance or complete absence of ColXVII on the cell surface has marked consequences for cell behaviour, i.e. it induces a nondirected migratory phenotype [6], [7]. Furthermore, ColXVII expression is increased in the epithelial tongue during the early phases of reepithelialization of acute wounds (own unpublished data) [8], [9] and in the invasive front of squamous cell carcinoma [10], [11]. However, the molecular mechanisms, which trigger these changes in cell motility remained elusive.

In this study we used murine *Col17a1* ***^−/^*** ^*−*^ keratinocytes to identify ColXVII dependent mechanisms in cell adhesion and migration. Our data unveiled an unexpected activation of phosphatidylinositol 3-kinase (PI3K) signaling via the β4 integrin subunit and the focal adhesion kinase (FAK) in the absence of ColXVII that resulted in Rac1 activation and less directed cell migration. Moreover, we demonstrated a link between ColXVII expression and linear cell migration, as overexpression of ColXVII in *Col17a1* ***^−/^*** ^*−*^ yielded in significantly increased directionality.

## Materials and Methods

### *Col17a1^−/−^* mice

The generation of the *Col17a1^−^* ^***/****−*^ mice has been described elsewhere [12]. Briefly, the targeting vector contained 6.7 kb genomic DNA with arms of 4.3 kb and 2.4 kb. Exon 18 and the surrounding intron sequences of the *Col17a1* gene were replaced by the neomycin resistance gene driven by a phosphoglycerate kinase promoter. Embryonic stem cell culture and the generation of chimeric mice were performed by the Biocenter Oulu Transgenic Core Facility. Chimeric mice were generated by blastocyst injection of embryonic stem cells carrying the targeted mutation and were mated with C57BL/6 J OlaHsd females to produce a targeted mouse line. F1 heterozygous mice were backcrossed for seven generations and then intercrossed to generate *Col17a1^−^* ^***/****−*^ mice. Studies using animal material were approved by the Animal Experiment Board of the University of Oulu as well as the National Animal Experiment Board of Finland (approval no. ESAVI/5255/04.10.03/2011).

### Keratinocyte cultures

Primary keratinocytes were prepared from newborn wild type and *Col17a1^−^* ^***/****−*^ mice as previously described [13]. Briefly, the epidermis was separated from the dermis by incubation with dispase overnight at 4°C. The epidermis was peeled off and incubated in trypsin/EDTA (0.5%/0.02%) solution in a shaker for 30 minutes at 37°C to release separated cells. The cell suspension was filtered through a 70 µm strainer and the cells were seeded in keratinocyte growth medium (CnT-07 medium, CellNTec, Bern, Switzerland) and incubated at 37°C, 5% CO_2_ and 95% humidity. Passage 0 to 2 keratinocytes were designated as primary wild type and *Col17a1^−^* ^***/****−*^ keratinocytes. To generate immortalized keratinocyte cell lines, the cells of different individuals were further separately cultured for two to three weeks, until several single colonies appeared. The colonies of each individual were then pooled which resulted in one cell line for each individual. Immortalized cells derived from at least six different individuals (matched wild type/*Col17a1^−^* ^***/****−*^ littermates from three different litters) were independently used for each experiment. Transformed keratinocytes isolated from wild type mice were referred to as control cells.

Primary human keratinocytes were obtained by trypsinization of control and JEB skin biopsies and cultured in serum-free, low-calcium keratinocyte growth medium supplemented with bovine pituitary extract and epidermal growth factor (Invitrogen, Karlsruhe, Germany) for 2 to 4 passages, as described before [14]. Studies using patient material were approved by the Ethics Committee of the University of Freiburg (approval no. 44/03) and conducted according to the Declaration of Helsinki. Patients provided written informed consent prior to their participation.

### β4 integrin knockdown

β4 integrins knockdown (kd) was generated using MISSION shRNA lentivirus (Sigma, München, Germany) according to the manufacturer's protocols. Briefly, *Col17a1^−^* ^***/****−*^ keratinocytes where seeded overnight in 6-well dishes, then infected with lentivirus encoding one of five different β4 integrin specific shRNAs with a multiplicity of 0.5 in culture medium supplemented with Polyprene (5 µg/ml). The following day, the medium was replaced with fresh medium containing puromycin (20 µg/ml) for selection of stable transfectans. Kd efficiency of the five different β4 integrin shRNAs was analyzed by Quantitative RT-PCR and two of them (kd#1 with 90% kd and kd#2 with 30% kd efficiency) have been selected for experiments.

### Collagen XVII overexpression

pcDNA3.1/myc-His(-) expression vectors encoding the cDNA sequence for murine full-length ColXVII [13] were used as templates to amplify the DNA by PCR using Phusion high-fidelity DNA polymerase (Fermentas, St. Leon-Rot, Germany). The following primers were used: forward primer containing a BglII restriction site: 5′gaagatctgctagccaccatggatgtgaagatct 3′ and the reverse primer containing a EcoRI restriction site: 5′gggaatcgctcaatgatgatgatgatgatg 3′. The resulting PCR product was ligated into a pJET1.2/blunt vector (Fermentas, St. Leon-Rot, Germany) following the manufacturer's instructions and transformed into competent *Escherichia coli* (DH5α). Purified DNA was digested with BglII and EcoRI restriction enzymes, gel purified and ligated into the retroviral MSCV-IRES-GFP (pMIG) expression vector using T4 DNA ligase (New England Biolabs, Frankfurt, Germany) and subsequently transformed into competent *Escherichia coli* (DH5α). Positive clones were confirmed by sequencing.

Amphotropic retroviruses were generated by co-transfecting retroviral ColXVII DNA and retroviral helper plasmids (pHit60 and pVSV-G) in a 1∶1 ratio into HEK293T cells using Superfect (Sigma München, Germany). Virus production was stimulated by sodium-butyrate [5 mM] treatment for 8 hours. 24 hours after stimulation the viral supernatants were collected and cleared by syringe-filtration (0.45 µm). Polybrene was added in a final concentration of 5 µg/ml.

*Col17a1^−^* ^***/****−*^ keratinocytes were transduced with retroviral particles for 24 hours. Thereafter, virus-containing medium was replaced by CnT-07 growth medium.

### Antibodies and reagents

The following monoclonal antibodies were used: mouse AC-15 to actin (Sigma, München, Germany), mouse B-1 to Akt1 (Santa Cruz, Heidelberg, Germany), rabbit D9E to phospho-Akt (S473) (Cell signaling technology, Danvers, USA), mouse 36/E-cadherin (BD Bioscience, Heidelberg, Germany), mouse 4.47 to phospho-FAK Y397 (Millipore, Darmstadt, Germany) and rat MB1.2 to β1 integrin (Merck, Darmstadt, Germany). The polyclonal antibodies were: rabbit ERK2 (C-14), rabbit GAPDH and rabbit integrin β4 (H-101) (Santa Cruz, Heidelberg, Germany), rabbit phospho-p44/42 MAPK (ERK1/2; T202/Y204) (Cell signaling technology, Danvers, USA), rabbit phospho-FAK (Y397) (Invitrogen, Karlsruhe, Germany), rabbit Keratin 5 (HISS Diagnostic, Freiburg, Germany) and the rabbit Endo-2 antibody to ColXVII [15].

A polyclonal rabbit antibody specific for the phosphorylated residue S1356 on the β4 integrin subunit was kindly provided by A. Sonnenberg (The Netherlands Cancer Institute) [16].

The inhibitor PI3K (LY294002) was obtained from Merck Biosciences (Darmstadt, Germany). Selective focal adhesion kinase (FAK) inhibitors PF 573228 and FAK Inhibitor 14 were obtained from R&D Systems (Wiesbaden-Nordenstadt, Germany). Recombinant EGF was obtained from ImmunoTools (Friesoythe, Germany). All inhibitors are used in indicated concentrations.

### Immunofluorescence microscopy

Keratinocytes were grown on cover-slips and fixed in methanol for 3 minutes, followed by acetone fixation for 30 seconds. Unspecific binding sites were blocked by incubation with 1% BSA/TBS and the incubation with the primary antibodies was overnight at 4°C, followed by secondary antibodies and nuclear staining with DAPI. The slides were mounted in Dako Fluorescence Mounting Medium, examined with a Axiophot 2E photomicroscope (Carl Zeiss, Germany) equipped with Zeiss Plan-Neofluar and Apochromat lenses 63x, NA 1.25 and 1.4 and recorded with a digital camera (AxiocamHR, Carl Zeiss, Germany). Image analysis and processing were performed using the AxioVision LE 4.6 (Carl Zeiss, Germany) and Adobe Illustrator Artwork CS4 software.

### Cell detachment assays

Two types of cell detachment assays were employed. For the trypsin based detachment assay, 4×10^4^ keratinocytes/well were seeded in 96-well plates, and after 24 hours, incubated with trypsin/EDTA (0.05/0.02%) for 0–10 minutes. For the centrifugal-force assay, 4×10^4^ keratinocytes were seeded onto 96-well plates coated with laminin 332 (1 µg/ml) (Millipore, Darmstadt, Germany). Cell adhesion was allowed to proceed for 10 minutes. Thereafter, the wells were completely filled with medium, sealed with Thermowell sealing tape and inverted before centrifugation in a tabletop centrifuge for 8 minutes at indicated forces. In both assays, non-adherent cells were removed by washing with PBS. Adherent cells were fixed with methanol and stained with 0.5% crystal violet in 20% (v/v) methanol. The dye was released from the cells by addition of 1% SDS, and the absorbance of the dye solution determined at 595 nm. The adhesion of the cells was expressed as a percentage relative to untreated controls (0 minutes trypsin/EDTA and 0×g respectively).

### Cell migration assays and cell tracking

For assessment of the motility of individual cells, 4×10^4^ keratinocytes were plated on uncoated 35 mm glass-bottom culture dishes (Ibidi, Munich, Germany), and the motility of the cells was monitored by the time-lapse imaging system Nikon's Biostation IM (Improvision, Coventry, UK). Phase-contrast photographs were captured every 5 minutes during 4 hours. Cell/nuclei tracking in two dimensions (x, y) was performed with Imaris 6.2.0 software (Imaris, Melville, NY). From these data the linear distance was calculated, defined as the ratio of the distance between the start and end position of the nucleus (track displacement length) to the total distance actually travelled by the cell (track length), which yielded the Processive Index (PI). The PI for each cell is shown as a function of the total distance travelled in µm.

### Quantitative RT-PCR

Total cellular RNA was extracted with RNeasy (Qiagen, Hilden, Germany). 1 µg of total RNA was reverse transcribed using a First Strand cDNA Synthesis kit (Fermentas, St. Leon-Rot, Germany). Relative quantification of gene expression was performed by real-time quantitative PCR using iQ SYBR-Green Supermix on the CFX96TM C1000TM Thermal Cycler (Bio-Rad, München, Germany) following the manufacturer's protocols. Relative gene expression was normalized to GAPDH expression.

### Protein extraction and immunoblotting

Back skin samples of mice (postnatal day 2 to 5) were homogenized in lysis buffer (0.1 M NaCl, 25 mM Tris-HCL pH 7.4, 1% Triton-X100, 0.1% SDS, Protease-Inhibitor Mix III (Merck Biosciences, Darmstadt, Germany), Phosphatase-Inhibitor cocktail 2 (Sigma, München, Germany), 1 mM Pefabloc and 2 mM EDTA) on ice with a T18 basic Ultra Turrax (Ika). Cultured keratinocytes were directly solubilized in lysis buffer and kept on ice for 1 hour.

The lysates were clarified by centrifugation at 10,000×g for 30 minutes at 4°C, and the protein concentration of the supernatant was determined using the protein quantification kit (Bio-Rad Laboratories). Aliquots containing equal amounts of protein were mixed with 4× concentrated SDS-PAGE sample buffer, boiled for 5 minutes and resolved on 10% SDS-PAGE under reducing conditions. For immunoblotting, the proteins were electro-transferred to 0.1 µm nitrocellulose membranes (GE Healthcare) by tank blotting using Towbin buffer [17]. The blots were incubated in 5% milk/TBS-Tween 0.1% containing the specific antibodies overnight at 4°C. The incubation with horseradish peroxidase-conjugated secondary antibodies was for 1 hour at room temperature. The detection was performed with the ECL Western blotting detection system (GE Healthcare, München, Germany). Immunoblot signals were densitometrically quantified with Fusion SL and BIO-1D Advanced software (PeqLab Biotechnologie GmbH, Germany).

### Rac G-LISA

Quantitative analysis of active Rac1 was performed using a G-LISA Rac activation assay (Cytoskeleton, Inc., Denver CO). Therefore, sub-confluent cells were lysed, and the protein concentration was determined using Precision RedTM Advanced Protein Assay Reagent. Samples at a concentration of 1 mg/ml were processed for the quantification of active Rac1 in a 96-well plate format according to the manufacturer's protocol. The signal intensity was determined by measuring absorbance at 490 nm using a microplate spectrophotometer.

### Statistical Analysis

All data are shown as mean ± SEM and statistical significance was determined using two-tailed Student's t-test. Calculations were performed with Prism version 5.0 (GraphPad Software). For all analyses differences are considered to be statistically significant at *p*<0.05.

## Results

### Lack of ColXVII in keratinocytes results in altered cell adhesion and spreading

**Figure 1:**
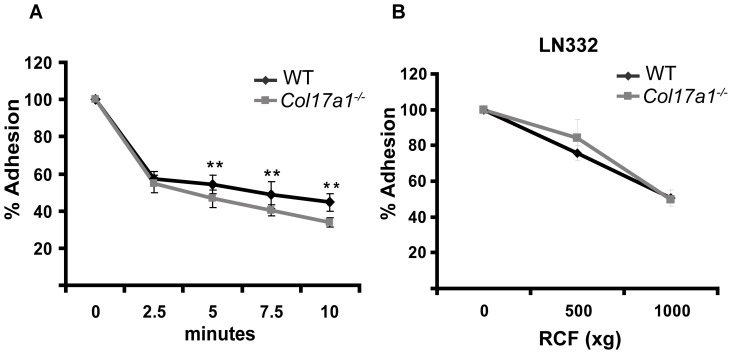
Altered cell detachment in the absence of ColXVII. **A**, Trypsin-based cell detachment assay. Confluent cell layers of keratinocytes derived from wild type (Ctrl) and *Col17a1^−^* ^***/****−*^ mice were treated with trypsin/EDTA (0.05%/0.02%) for indicated time points (cells of three individuals per genotype have been analyzed; number of independent measurements  = 5). **B**, For the centrifugal force-based cell detachment assay cells were allowed to adhere on laminin 332 (LN332) for 10 minutes before measuring the strength of the adhesion at indicated centrifugal forces (cells of three individuals per genotype have been analyzed; number of independent measurements  = 3). For both assays adherent cells were stained with 0.5% crystal-violet, lysed with 1% SDS, and the percentage of adherent cells was determined spectrophotometrically at 540 nm. Data are shown as mean ± SEM; **p<0.01.

**Figure 2:**
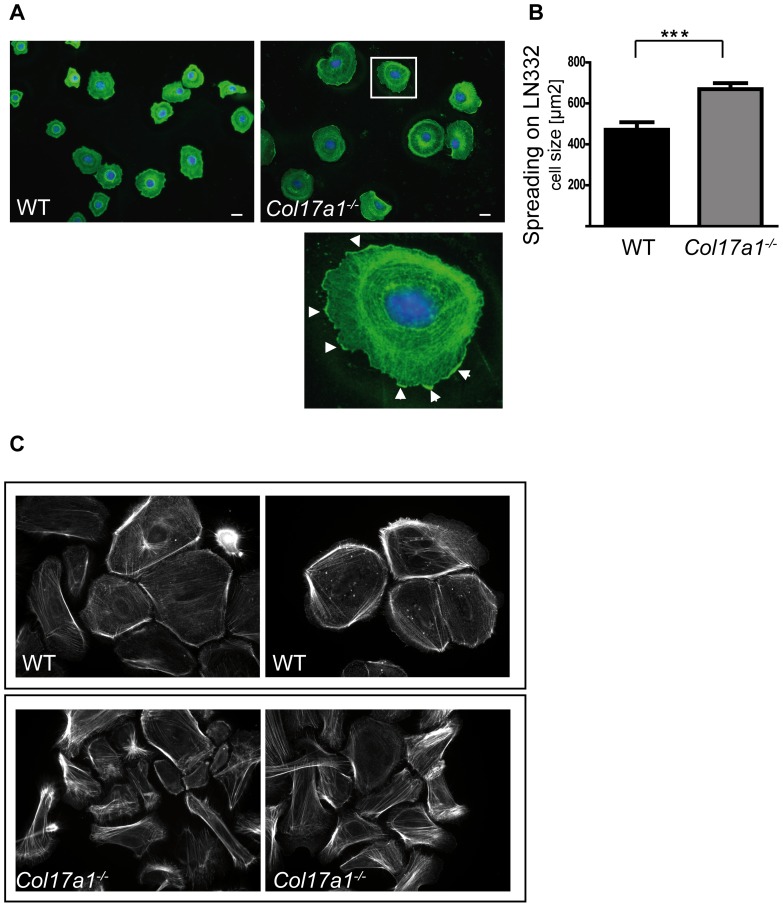
Enhanced spreading and actin dynamics in *Col17a1^−/−^* keratinocytes. **A**, Keratinocytes derived from wild type (Ctrl) and *Col17a1^−^* ^***/****−*^ mice were grown on LN332 coated chamber slides for 30 minutes, fixed and processed for indirect immunofluorescence staining with an actin antibody. The insert represents one enlarged *Col17a1^−^* ^***/****−*^ cell. Arrowheads indicate the presence of multiple lamellipodia visible by tightly dense actin staining. *Scale bar  = 10 µm*. **B**, The graph shows the cell size in µm^2^. The size of *Col17a1^−^* ^***/****−*^ keratinocytes was about 40% larger than that of Ctrl cells (*n = 30*; cells of two individuals per genotype have been analyzed). Data are shown as mean ± SEM; ***p<0.001. **C**, Semi-confluent Ctrl and *Col17a1^−^* ^***/****−*^ keratinocytes were fixed with 4% PFA and incubated with rhodamin-conjugated phalloidin for 1 hour. Phalloidin-staining indicated a higher number of stress fibers and reduced cortical actin in *Col17a1^−^* ^***/****−*^ keratinocytes.

Human diseases and mouse models have demonstrated that loss of ColXVII expression is associated with rudimentary poorly developed HDs and separation of the epidermis from the basement membrane upon mechanical friction [12], [18], [19]. Here we assessed the adhesive strength of murine *Col17a1^−^* ^***/****−*^ keratinocytes using two different *in vitro* detachment assays. One was the trypsin/EDTA-induced detachment of keratinocytes, which measures the strength of cell attachment to own ECM established over 24 hours, and the other was centrifugal force-induced detachment, which measures the adhesion strength of keratinocytes that have been allowed to attach on LN332 for 10 minutes. After 10 minutes treatment with trypsin/EDTA, 49.5 (±4.5) % of control cells, but only 31.5 (±2.5) % of the *Col17a1^−^* ^***/****−*^ keratinocytes remained attached to the plate (Figure 1A). However, the centrifugal force assay as direct measure for cell adhesive strength revealed no difference when the cells were seeded on LN332 (Figure 1B). In contrast, the cell spreading ability of *Col17a1^−^* ^***/****−*^ keratinocytes on LN332 exhibited differences. *Col17a1^−^* ^***/****−*^ keratinocytes showed enhanced spreading (Figure 2A and B) and formation of multiple lamellipodia (Figure 2A, insert), indicating propensity to migrate. Phalloidin-staining indicated a higher number of stress fibers and reduced cortical actin in *Col17a1^−^* ^***/****−*^ keratinocytes (Figure 2C), revealing elevated dynamics of the actin cytoskeleton and enhanced disposition to cell motility.

**Figure 3:**
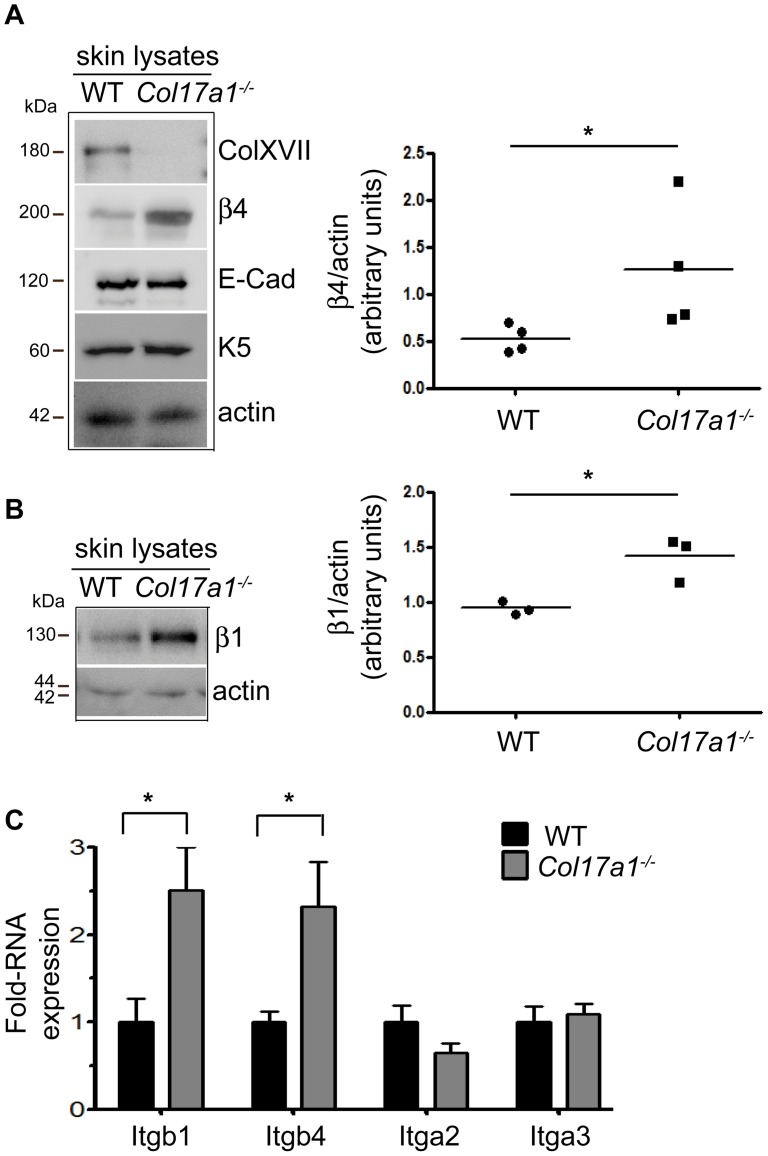
Upregulation of α6β4 and β1 integrins in *Col17a1^−/−^* skin. **A** and **B**, Skin lysates from WT and *Col17a1^−^* ^***/****−*^ mice were immunoblotted with indicated antibodies. The graphs combine the quantification for β4 protein expression of four individuals per genotype (**A**) and β1 protein expression of three individuals (**B**). *p<0.05. **C**, Quantitative RT-PCR of primary wild type and *Col17a1^−^* ^***/****−*^ keratinocytes (cells of four individuals per genotype have been analyzed; number of independent measurements  = 3). Data are shown as mean ± SEM; *p<0.05.

**Figure 4:**
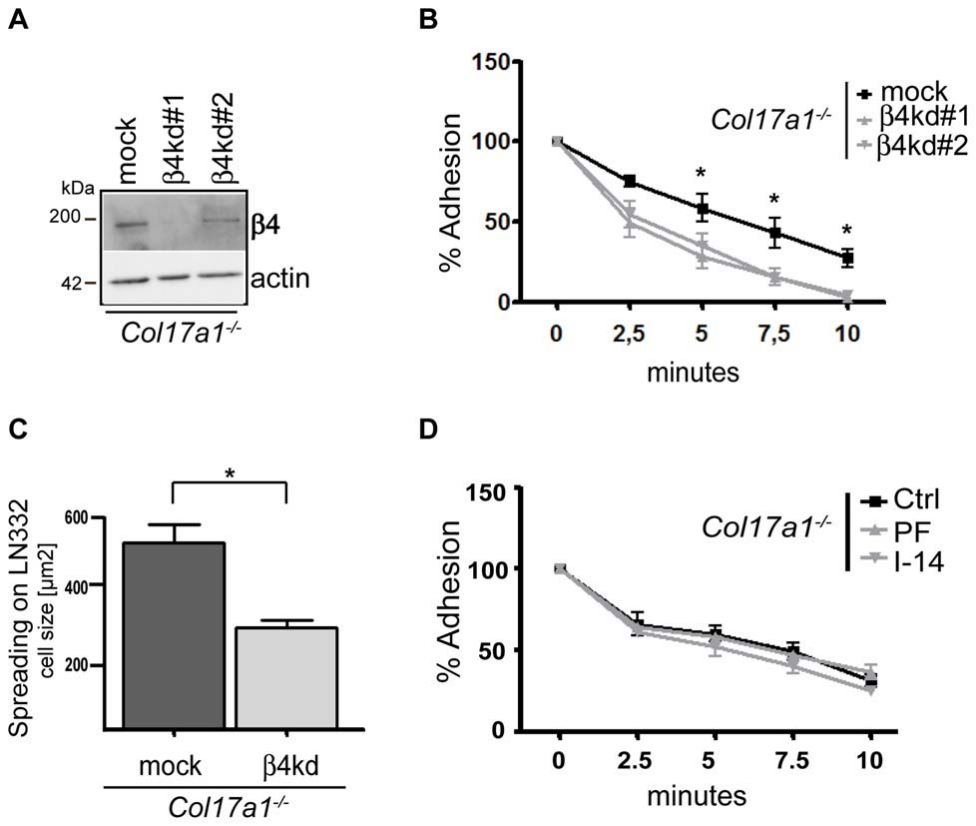
β4 integrin subunit is functionally involved in cell adhesion and spreading of *Col17a1^−/−^* cells. *Col17a1^−^* ^***/****−*^ keratinocytes were transduced with empty pLKO vector (mock) and two different shRNA to β4 integrin subunit (β4kd#1 and β4kd#2). **A**, The cells were lysed and equal amounts of total protein were immunoblotted with indicated antibodies. **B**, Confluent layers of *Col17a1^−^* ^***/****−*^ cells were subjected to trypsin/EDTA detachment assay (as described in Figure 1). The percentage of adherent cells are shown as mean ± SEM (number of independent measurements  = 3); *p<0.05. **C**, Cells were grown on LN332 coated chamber slides for 30 minutes, fixed and processed for indirect immunofluorescence with an actin antibody. The graph shows the cell area in µm^2^ (*n = *35). The data are shown as mean ± SEM. *p<0.05. **D**, *Col17a1^−^* ^***/****−*^ keratinocytes were treated with DMSO (Ctrl) or different phospho-FAK inhibitors (PF 573228 [5 µM] and Inhibitor 14 [1 µM]) for 6 hours and thereafter subjected to trypsin/EDTA detachment assay. The data are shown as mean ± SEM (cells of three individuals have been analyzed; number of independent measurements  = 5).

The adherence of keratinocytes to the extracellular matrix is mainly mediated by two different cell-matrix junctions namely the hemidesmosomes and focal adhesions. Both multi-protein complexes mediate cell-matrix adhesion via distinct integrin heterodimers, like the hemidesmosomal α6β4 integrin as well as the focal adhesion integrins α2β1and α3β1. Ligation of these integrins integrates a link to the keratin or actin cytoskeleton thereby regulating cell adhesion, spreading and migration. Interestingly, the protein levels of β4 and β1 integrin subunits were significantly induced in *Col17a1^−^* ^***/****−*^ skin (Figure 3A and B) while the adherens junction protein E-Cadherin and the cytokeratin 5 remained unchanged. Further analysis also revealed strongly increased transcription levels of *Itgb4* and *Itgb1* genes in primary keratinocytes (Figure 3C) as well as in spontaneously immortalized high-passage keratinocytes (Figure S1A). In contrast the transcription levels of *Itga2* and *Itga3* were unchanged (Figure 3C). These results imply that the increased α6β4 integrin production in the *Col17a1^−^* ^***/****−*^ keratinocytes may result in enhanced cell adhesion, especially on LN332. This assumption was confirmed by the lentiviral knockdown of the β4 integrin chain (β4kd) in *Col17a1^−^* ^***/****−*^ keratinocytes using two different β4 integrin specific shRNAs (Figure 4A). The trypsin/EDTA detachment assay revealed significant reduction of adhesion in these cells (Figure 4B) when compared to their respective parental *Col17a1^−^* ^***/****−*^ keratinocytes. In addition, β4 integrin subunit knockdown in *Col17a1^−^* ^***/****−*^ keratinocytes reduced spreading on LN332 by about 45% (Figure 4C).

Keratinocyte adhesion also depends on the dynamic turnover of focal adhesions, which are regulated by outside-in signaling of integrins α2β1 and α3β1 including the recruitment and auto-phosphorylation of focal adhesion kinase (FAK) at residue Y397 [20]. Golubovskaya et al. [21] demonstrated that inhibition of FAK auto-phosphorylation led to decreased β1 integrin subunit-mediated cell adhesion in a dose-dependent manner. Therefore, we used two different phospho-FAK inhibitors (Inhibitor 14 and PF 573228) to interfere the β1 integrin subunit-mediated cell adhesion. While both phospho-FAK inhibitors enhanced the cell detachment of control cells to their own matrix (Figure S2A), neither of them influenced the self-established adhesion of *Col17a1^−^* ^***/****−*^ keratinocytes (Figure 4D).

Taken together, these investigations highlight the elevated α6β4 integrin level as characteristic adhesion feature of *Col17a1^−^* ^***/****−*^ keratinocytes.

### *Col17a1^−/−^* keratinocytes show increased, but less directed motility

**Figure 5:**
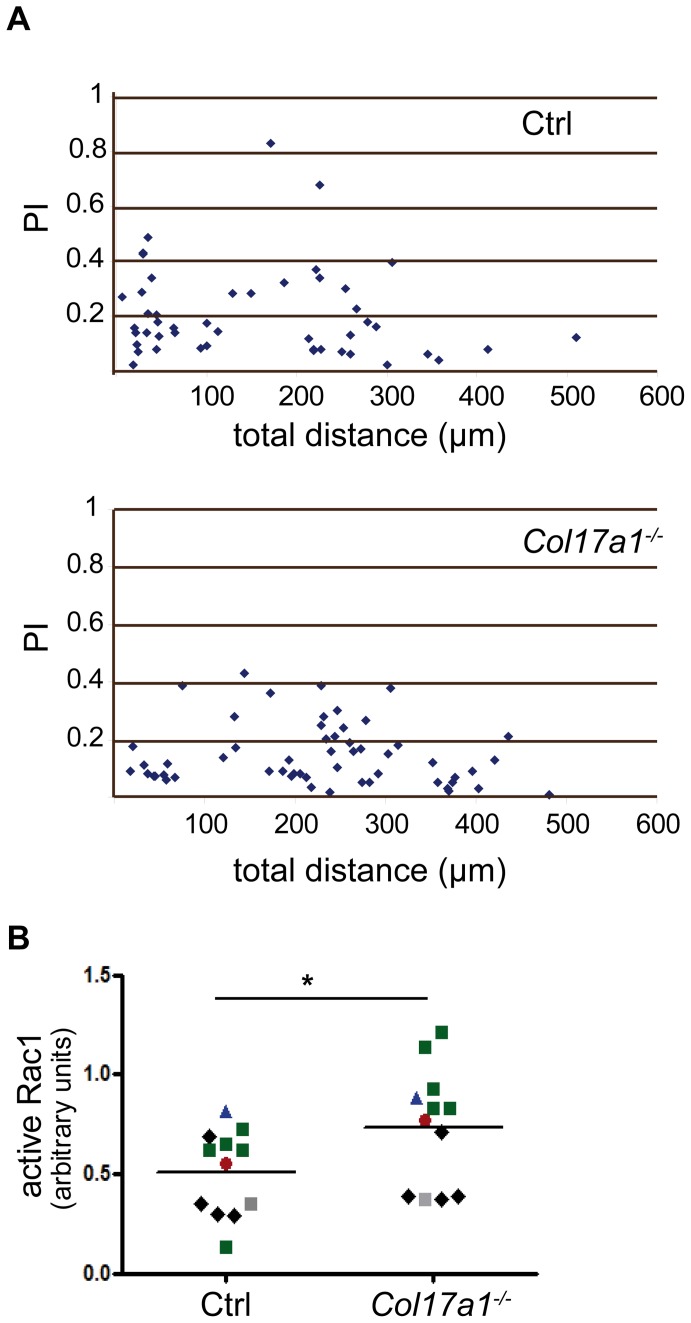
Motility of *Col17a1^−/−^* keratinocytes is enhanced via active Rac1. **A**, Keratinocytes derived from wild type (Ctrl) and *Col17a1^−^* ^***/****−*^ mice were grown on glass-bottom culture dishes, and cell migration was recorded by time-lapse imaging. The distance migrated is indicated on the x-axis. The y-axis indicates the processive index (PI) which is defined as the ratio of the distance between the start and end position of a cell to the total distance actually travelled by the cell. **B**, G-LISA was used to determine Rac1 activity in protein extracts of sub-confluent Ctrl and *Col17a1^−^* ^***/****−*^ keratinocytes. Equal amounts of total protein were used. Symbols indicate the matched Ctrl/*Col17a1^−^* ^***/****−*^ littermates (number of independent measurements  = 4); *p<0.05.

Since the expression of ColXVII is strongly elevated on the base of the epithelial tongue during reepithelialization of acute wounds (own unpublished data) [9] and at the invasive front of squamous cell carcinoma [10], [11], we performed cell migration assays using control and *Col17a1^−^* ^***/****−*^ keratinocytes. Tracking of individually migrating cells revealed that *Col17a1^−^* ^***/****−*^ keratinocytes migrate significantly longer distances, but in a less directed manner as shown by the low mean processive index (PI) of *Col17a1^−^* ^***/****−*^ keratinocytes (0.14±0.01) compared to that of control cells (0.20±0.02) (Figure 5A and Table 1). To assess whether ColXVII expression directly effects cell migration we performed a gain-of-function experiment by retroviral over-expression of ColXVII in *Col17a1^−^* ^***/****−*^ keratinocytes. Interestingly, this experiment resulted in a significantly increased processive index (PI) of these cells (Table 1), suggesting an important role of ColXVII especially in the regulation of the directional migration.

**Table 1:** Migration properties of control (Ctrl) and *Col17a1^−^* ^***/****−*^ keratinocytes (see also Figure 5A, 6B and S1B).

	Treatment	Mean total distance (µm)	Mean PI
	untreated	**156.4±18.3 (##)**	**0.20±0.02 (#)**
**Ctrl**	DMSO	158.0±29.9	0.20±0.03
	LY294002	105.9±14.9	0.26±0.03
	untreated	**224.4±15.8 (##)**	**0.14±0.01 (#)**
***Col17a1 ^−/−^***	DMSO	*194.4±22.7(§§)*	0.14±0.03
	LY294002	*77.9±13.6 (§§)*	0.14±0.02
***Col17a1 ^−/−^***	mock	152.2±42.1	*0.12±0.06 (§)*
	ColXVII	88.6±12.0	*0.45±0.06 (§)*

PI  =  processive index. The data are shown as mean ± SEM.

**(##)  =** *p<*0.01; **(#)  =** *p*<0.05; *(§§)  =  p<*0.001; *(§)*  =  *p*<0.01.

The modulation of the actin cytoskeleton and the formation of lamellipodia in migrating cells require Rac1 activation [22], [23]. The determination of lamellipodia formation and Rac1 activation in migrating *Col17a1^−^* ^***/****−*^ keratinocytes revealed multiple lamellipodia and accelerated Rac1 activity (Figure 5B), which indicates increased migratory activity in these cells.

### Increased PI3K activation in *Col17a1^−/−^* keratinocytes accelerates migration speed

**Figure 6:**
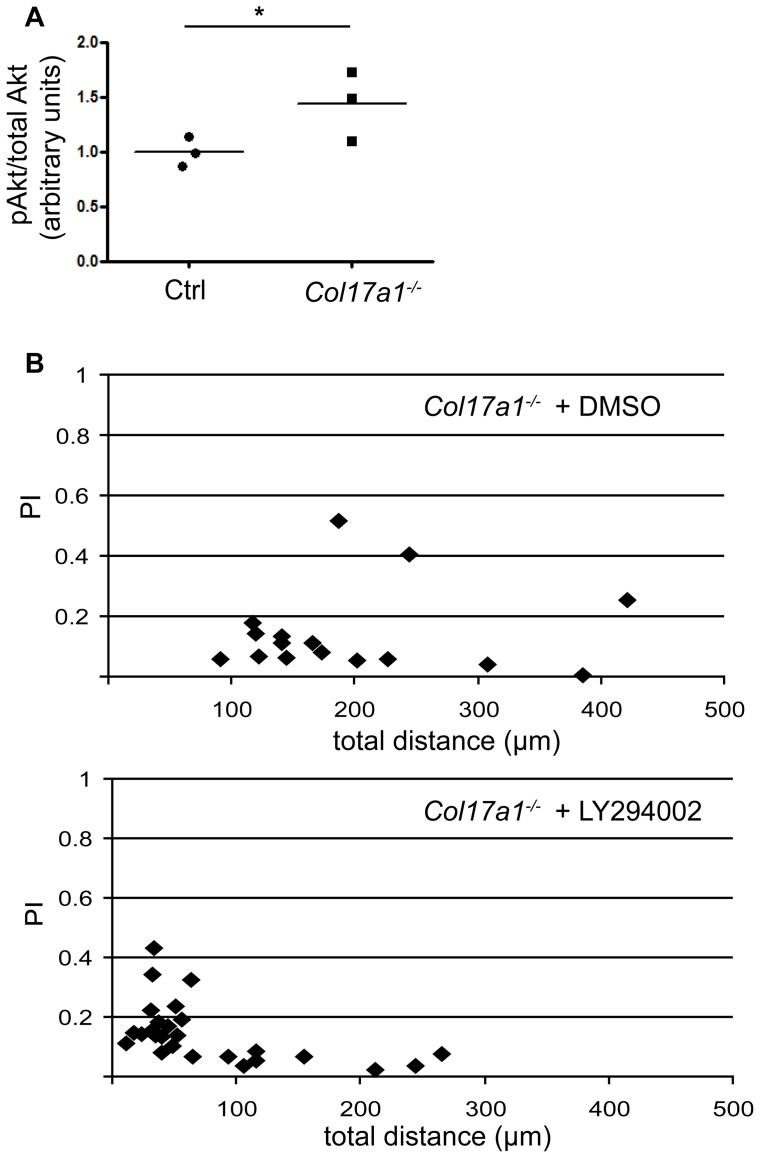
Enhanced PI3K signaling in *Col17a1^−/−^* keratinocytes. **A**, Keratinocytes derived from wild type (Ctrl) and *Col17a1^−^* ^***/****−*^ mice were lysed and equal amounts of total protein were immunoblotted with phospho-Akt and total Akt antibodies. The graph shows quantification of phospho-Akt relative to total Akt (cells of three individuals per genotype have been analyzed in 3 independent measurements); *p<0.05. **B**, *Col17a1^−^* ^***/****−*^ keratinocytes were grown on glass-bottom culture dishes and treaded with either DMSO or LY294002 [50 µM]. Cell migration was recorded by time-lapse imaging every 5 minutes during 4 hours. The distance migrated is indicated on the x-axis, the processive index (PI) on the y-axis.

The lipid kinase member phosphoinositide 3-kinase (PI3K) plays a pivotal role in cell migration, presumably because it regulates actin dynamics via Rac1 [24]–[26]. Consequently, it was of interest whether PI3K signaling is altered in *Col17a1^−^* ^***/****−*^ keratinocytes. Indeed, as an indicator of induced PI3K activity in these cells, phosphorylation of Akt at residue S473 was significantly increased (Figure 6A). To elucidate the contribution of PI3K in cell migration, *Col17a1^−^* ^***/****−*^ keratinocytes were treated with the selective PI3K inhibitor LY294002. The PI3K inhibition led to significantly decreased migration speed of *Col17a1^−^* ^***/****−*^ keratinocytes as documented by about 60% reduced migration distance compared to vehicle treated cells (Figure 6B, Table 1), while the same treatment on control keratinocytes did not lead to significant changes in their motility (Figure S1B, Table 1). In addition, the pharmacologic inhibition of PI3K showed no effect on the directionality of neither *Col17a1^−^* ^***/****−*^ keratinocytes nor control keratinocytes (Figure 6B, Figure S1B, Table 1).

### α6β4 integrin and phospho-FAK signaling contribute to PI3K activation in *Col17a1^−/−^* keratinocytes

The above data suggest that the ColXVII molecule may act as a repressor of PI3K signaling, which in turn seems to be important for the increase in keratinocyte motility. These findings led us to identify the upstream activators of PI3K in *Col17a1^−^* ^***/****−*^ keratinocytes. One important group of possible PI3K activators in keratinocytes are integrins, among those the ColXVII binding partner α6β4 integrin, as well as the α2β1 and α3β1 integrins represent good candidates.

The phosphorylation of different serine-residues within the cytoplasmic domain of the β4 integrin chain has been demonstrated to be involved in the induction of PI3K signaling [27]. It has been shown that phosphorylated α6β4 integrins dissociate from stable HDs and accumulate preferentially in F-actin rich cell protrusions [28]. To determine whether the β4 integrin subunit contributes to the PI3K activation in the absence of ColXVII expression, we analyzed its phosphorylation in human *COL17A1^−^* ^***/****−*^ keratinocytes derived from JEB patients, since only anti-human phospho-β4 integrin antibodies were available to us.

**Figure 7:**
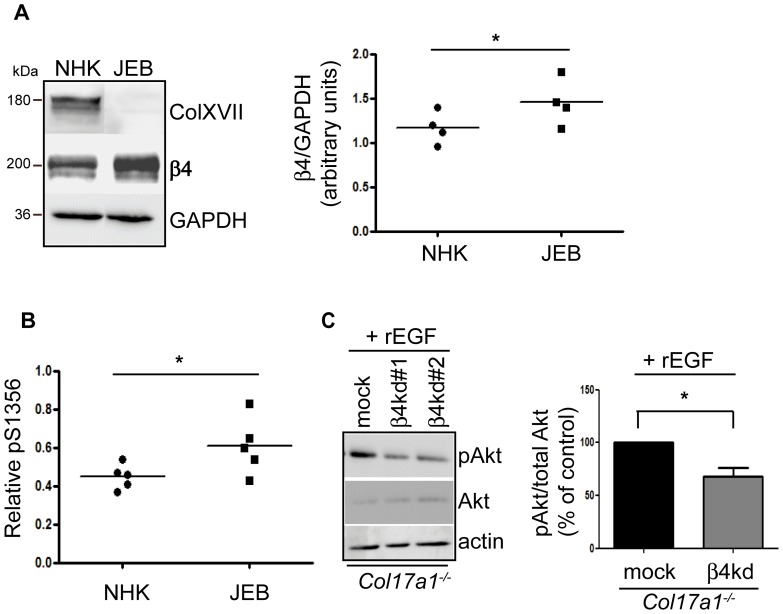
Loss of ColXVII leads to EGF-induced S1356 phosphorylation of the β4 integrin subunit. **A**, Normal human keratinocytes (NHK) and ColXVII-deficient JEB keratinocytes were lysed and equal amounts of total protein were immunoblotted with the indicated antibodies. The graph combines the quantification of NHKs (four donors) and JEB keratinocytes (four patients). *p<0.05. **B**, This graph shows the quantification of β4 integrin subunit S1356 phosphorylation in NHKs and ColXVII-deficient JEB keratinocytes relative to total β4 integrin subunit expression levels. *p<0.05. **C**, Sub-confluent *Col17a1^−^* ^***/****−*^ keratinocytes with β4 integrin knockdown (β4 kd#1 and β4 kd#2) were treated with recombinant EGF [200 ng/ml] for 10 minutes, lysed and immunoblotted with indicated antibodies. Quantification is shown as mean ± SEM (number of independent measurements  = 3, including two clones with β4 knockdown and a mock control); *p<0.05.

Human *COL17A1^−^* ^***/****−*^ keratinocytes seemed very similar to their murine counterparts, they also showed increased *ITGB4* gene activity (not shown) and β4 integrin protein expression (Figure 7A). Interestingly, human *COL17A1^−^* ^***/****−*^ keratinocytes showed significantly induced activation of the β4 integrin subunit as demonstrated by elevated S1356 phosphorylation (Figure 7B), which uncovers β4 integrin cytotail signaling as an activator of PI3K in *Col17a1^−^* ^***/****−*^ keratinocytes.

It has been demonstrated that EGF-induced phosphorylation of the β4 integrin subunit can subsequently activate PI3K in keratinocytes [16], [26], [28]–[32]. This prompted us to determine PI3K activity in murine *Col17a1^−^* ^***/****−*^ keratinocytes with β4 integrin subunit knockdown. Indeed, these cells revealed a significant decrease of PI3K activity in the presence of recombinant EGF as demonstrated by about 30% reduced phosphorylation of Akt (Figure 7C), indicating that PI3K activation in *Col17a1^−^* ^***/****−*^ keratinocytes partially depends on the β4 integrin subunit.

**Figure 8:**
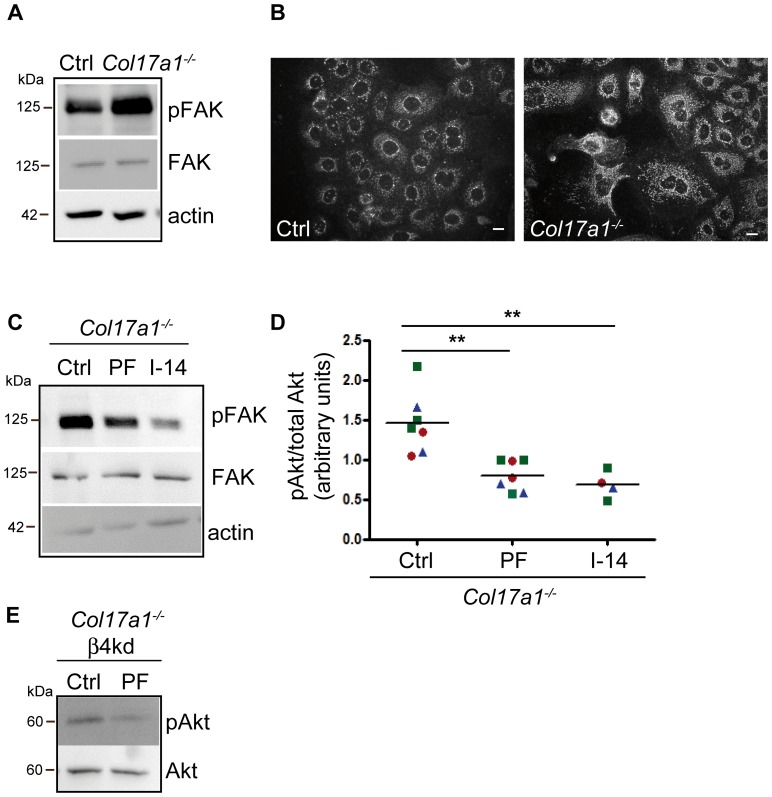
Loss of ColXVII leads to phosphorylation of FAK. **A**, Keratinocytes derived from wild type (Ctrl) and *Col17a1^−^* ^***/****−*^ mice were allowed to adhere to LN332 for 2 hours, lysed and immunoblotted with antibodies to phospho-FAK (Y397) and total FAK. **B**, Indirect immunofluorescence staining of Ctrl and *Col17a1^−^* ^***/****−*^ keratinocytes with phospho-FAK (Y397) antibody. *Scale bar  = 10 µm*. **C** and **D**, *Col17a1^−^* ^***/****−*^ keratinocytes were treated with DMSO (Ctrl) and two different phospho-FAK inhibitors (PF 573228 [5 µM] and Inhibitor 14 [1 µM]) for 6 hours. The cells were lysed and equal amounts of total protein were immunoblotted with antibodies to phospho-FAK (Y397) and total FAK (**C**, representative immunoblot) and to phospho-Akt and total Akt (**D**). The graph shows quantification of phospho-Akt relative to total Akt. Individuals are indicated by different symbols (number of independent measurements  = 2); **p<0.01. **E**, *Col17a1^−^* ^***/****−*^ keratinocytes with comprising β4 integrin subunit knockdown were treated with DMSO (Ctrl) or phospho-FAK inhibitor (PF 573228 [5 µM] for 6 hours. The cells were lysed and equal amounts of total protein were immunoblotted with antibodies to phospho-Akt and total Akt.

The increase in β1 integrin subunit expression in the epidermis of *Col17a1^−^* ^***/****−*^ skin (Figure 3B) was associated with enhanced phosphorylation of FAK (Figure 8A) in *Col17a1^−^* ^***/****−*^ keratinocytes seeded on LN332, which appeared along the highly enriched stress fibers (Figure 8B). In contrast, there was no difference in FAK phosphorylation between *Col17a1^−/−^* and control cells when seeded on fibronectin or collagen I (Figure S2B). These findings indicate accelerated dynamics in the assembly and disassembly of focal adhesions in *Col17a1^−^* ^***/****−*^ keratinocytes and mirror characteristics of highly motile cells. The involvement of phospho-FAK in PI3K activation was assessed using two different FAK inhibitors, both of them targeting selectively the auto-phosphorylation at Y397 upon integrin-matrix ligation (Figure 8C).

Prevention of FAK autophosphorylation in control cells using the FAK Inhibitor PF 573228 revealed a reduction in Akt phosphorylation (Figure S2C). Likewise, the addition of either FAK Inhibitor 14 or PF 573228 to *Col17a1^−^* ^***/****−*^ keratinocytes significantly reduced the phosphorylation of Akt (Figure 8D). This effect was even increased in *Col17a1^−^* ^***/****−*^ cells with β4 integrin subunit knockdown (Figure 8E), indicating the contribution of phospho-FAK in the regulation of PI3K activity in *Col17a1^−^* ^***/****−*^ keratinocytes besides α6β4 integrin signaling.

In summary, our results suggest that both, α6β4 integrin and phospho-FAK mediated signaling contribute to the increased activation of PI3K in *Col17a1^−^* ^***/****−*^ keratinocytes.

## Discussion

The role of the epidermal adhesion molecule ColXVII in cell migration was initially described in primary keratinocytes derived from JEB patients, demonstrating that low abundance or complete absence of ColXVII on the cell surface resulted in increased, but nondirected motility [6], [7]. In agreement, our *in vitro* investigations on murine *Col17a1^−^* ^***/****−*^ keratinocytes demonstrated the same negative correlation between the expression of ColXVII and the speed/directionality of cell migration. In contrast, viral knockdown of ColXVII in epithelial cell lines by siRNA or shRNA approaches disclosed a positive correlation between ColXVII expression and migration speed [33], [34]. These controversial results could be explained by differences in the compensatory effects of genetic cell models and virally transduced cell systems. For example the expression of the β4 integrin subunit was strongly upregulated in *Col17a1^−^* ^***/****−*^ keratinocytes, but unaffected by viral ColXVII knockdown in HEK cells [34]. The upregulation of the β4 integrin subunit in murine and human ColXVII deficient keratinocytes seems to be an important driver for the formation of multiple, but unstable lamellipodia, which results in less directed cell migration compared to control cells. Furthermore, we provide evidence for accelerated EGF-induced β4 integrin subunit phosphorylation and the subsequent activation of PI3K/Rac1 signaling in the absence of ColXVII. In line with this, the formation of lamellipodia during carcinoma invasion has been linked to the α6β4/PI3K/Rac1 pathway [24]–[26], [35].

There are two major lines of evidence to suggest that ColXVII regulates cell motility via enhanced phosphorylation of the α6β4 integrin. The first relates to different dynamic stages of the two types of HDs. ColXVII is present only in type I HDs, and its lack in type II HDs is compatible with cell migration. For example, intestinal cells that physiologically migrate along the villi have abundant type II, but no type I HDs [36], and COS-7 cells engineered to generate type II HDs do not curtail their migration [32]. Interestingly, phosphorylation of the β4 integrin cytotail is found predominantly in the more dynamic type II HDs, indicating that β4 integrin phosphorylation may prevent its interaction with ColXVII [28]. The second line of evidence stems from migrating carcinoma cells in which α6β4 integrin disappears from stable HDs and accumulates preferentially in F-actin rich cell protrusions [28], [37]. This process requires phosphorylation of the intracellular β4 domain and does not allow the binding of the ColXVII endodomain, indicating that the phosphorylation regulates the binding of ColXVII to β4 integrin subunits. Therefore, it seems likely that the absence of ColXVII favors phosphorylation of the β4 integrin cytotail.

Based on the above observations we conclude that enhanced PI3K signaling most likely determines the speed of migrating *Col17a1^−^* ^***/****−*^ keratinocytes. However, our data suggests that the directionality of the keratinocytes strongly depends on the presence of ColXVII. This would also explain the importance of an enhanced expression of ColXVII in keratinocytes during reepithelialization of acute wounds [9] and at the invasive front of squamous cell carcinoma [10], [11]. The present data support the working model of Tsuruta et al. [2] which suggests that only the presence of α6β4 integrin together with ColXVII/BP230 and the actin cytoskeleton can stabilize an extending lamellipodium and support directed cell migration.

Recently, it was suggested that the α6β4 integrin acts as a master regulator of α2β1 and α3β1 integrin transcription and expression via PI3K/mTOR pathway in keratinocytes [38]. These two laminin- and collagen-binding integrins are, in turn, regulators of focal adhesion dynamics in keratinocytes [31]. Integrin-linked focal adhesions are complexes that provide transient anchoring points for migrating cells. Upon cell-matrix adhesion, the integrins become activated and cluster in the plasma membrane. As an intracellular consequence FAK is recruited and rapidly autophosphorylated. At this stage phospho-FAK can promote cell migration in two ways; first by binding to PI3K and thereby activating signaling and, second, by influencing the remodelling of the actin cytoskeleton through the regulation of Rho GTPases [22]. As shown in the present study, *Col17a1^−^* ^***/****−*^ keratinocytes display enhanced expression of the β1 integrin subunit, elevated levels of phospho-FAK and increased stress fibers accompanied by reduced cortical actin. Interference of the phospho-FAK/PI3K pathway with selective FAK inhibitors revealed reduced phospho-Akt levels, suggesting that phospho-FAK might contribute to the β4 integrin-dependent PI3K activation in the absence of ColXVII. However, further studies examining the degree of β1 integrin subunit signaling in *Col17a1^−^* ^***/****−*^ keratinocytes are warranted.

**Figure 9:**
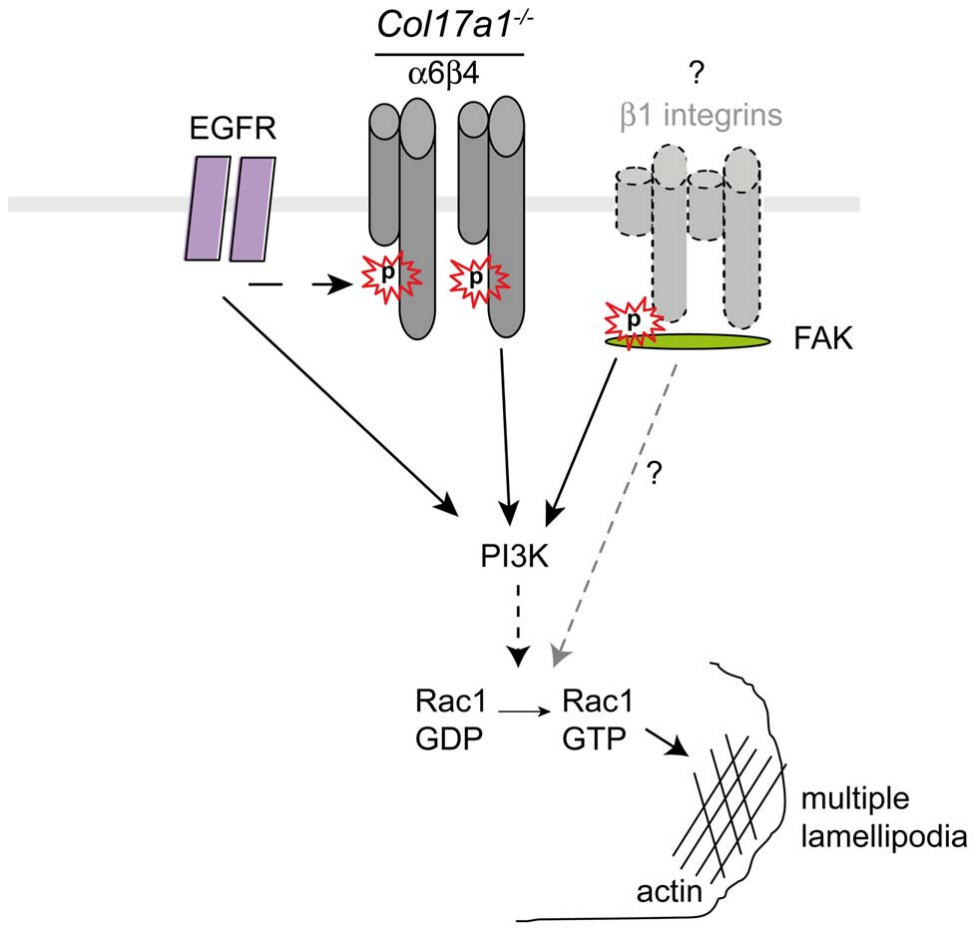
A schematic model of the migratory phenotype of *Col17a1^−/−^* keratinocytes. In wild type cells the endodomain of ColXVII binds to the intracellular domain of the β4 integrin subunit. In *Col17a1^−^* ^***/****−*^ keratinocytes, the genetic ablation of ColXVII leads to increased expression and phosphorylation (S1356) of the β4 subunit and to phosphorylation of FAK (Y397), which, in turn, enhance PI3K activity and induce undirected cell migration via Rac1 activation.

In summary, we used both human and murine ColXVII-deficient keratinocytes to provide evidence that cell adhesion, spreading and migration are modulated by synergistic actions of the phosphorylated β4 integrin subunit and phospho-FAK. These upstream activators of PI3K also effectuate activation of Rac1 (Figure 9). High levels of active Rac1 and the β4 integrin subunit initiate the formation of multiple unstable lamellipodia, which result in less directed cell migration. These findings provide mechanistic evidence that ColXVII represents an important coordinator of keratinocyte adhesion and directed motility by dampening integrin dependent PI3K activation and by stabilizing lamellipodia. These effects may contribute to the leading edge formation in acute wounds and invasive squamous cell carcinoma.

## Supporting Information

Figure S1 **A,** Quantitative RT-PCR of immortalized (high passage) control and *Col17a1^−^* ^***/****−*^ keratinocytes (cells of four individuals per genotype have been analyzed; number of independent experiments  = 3). **B,** Keratinocytes derived from wild type (Ctrl) mice were grown on glass-bottom culture dishes and treated with either DMSO or LY294002 [50 µM]. Cell migration was recorded by time-lapse imaging every 5 minutes during 4 hours. The distance migrated is indicated on the x-axis, the processive index (PI) on the y-axis.
(TIF)

Figure S2 **A**, Keratinocytes derived from wild type (Ctrl) mice were treated with DMSO or different phospho-FAK inhibitors (PF 573228 [5 µM] and Inhibitor 14 [1 µM]) for 6 hours and thereafter subjected to the trypsin/EDTA detachment assay. The data are shown as mean ± SEM (cells of three individuals have been analyzed; number of independent measurements  = 3). **B**, Keratinocytes derived from wild type (Ctrl) and *Col17a1^−^* ^***/****−*^ mice were allowed to adhere to fibronectin (FN) and collagen I (Col I) for 2 hours, lysed and immunoblotted with antibodies to phospho-FAK (Y397), total FAK and actin. **C**, Keratinocytes isolated from wild type mice (Ctrl) were treated with DMSO or different phospho-FAK inhibitors (PF 573228 [5 µM] and Inhibitor 14 [1 µM]) for 6 hours and thereafter subjected to trypsin/EDTA detachment assay. The data are shown as mean ± SEM (cells of three individuals have been analysed, number of independent measurements  = 3); *p<0.05.
(TIF)
