# Supplementary figures and images for: Transmembrane Collagen XVII Modulates Integrin Dependent Keratinocyte Migration via PI3K/Rac1 Signaling
Source: PLoS One. 2014 Feb 5;9(2):e87263. doi: 10.1371/journal.pone.0087263 (PMC3914815; doi:10.1371/journal.pone.0087263)

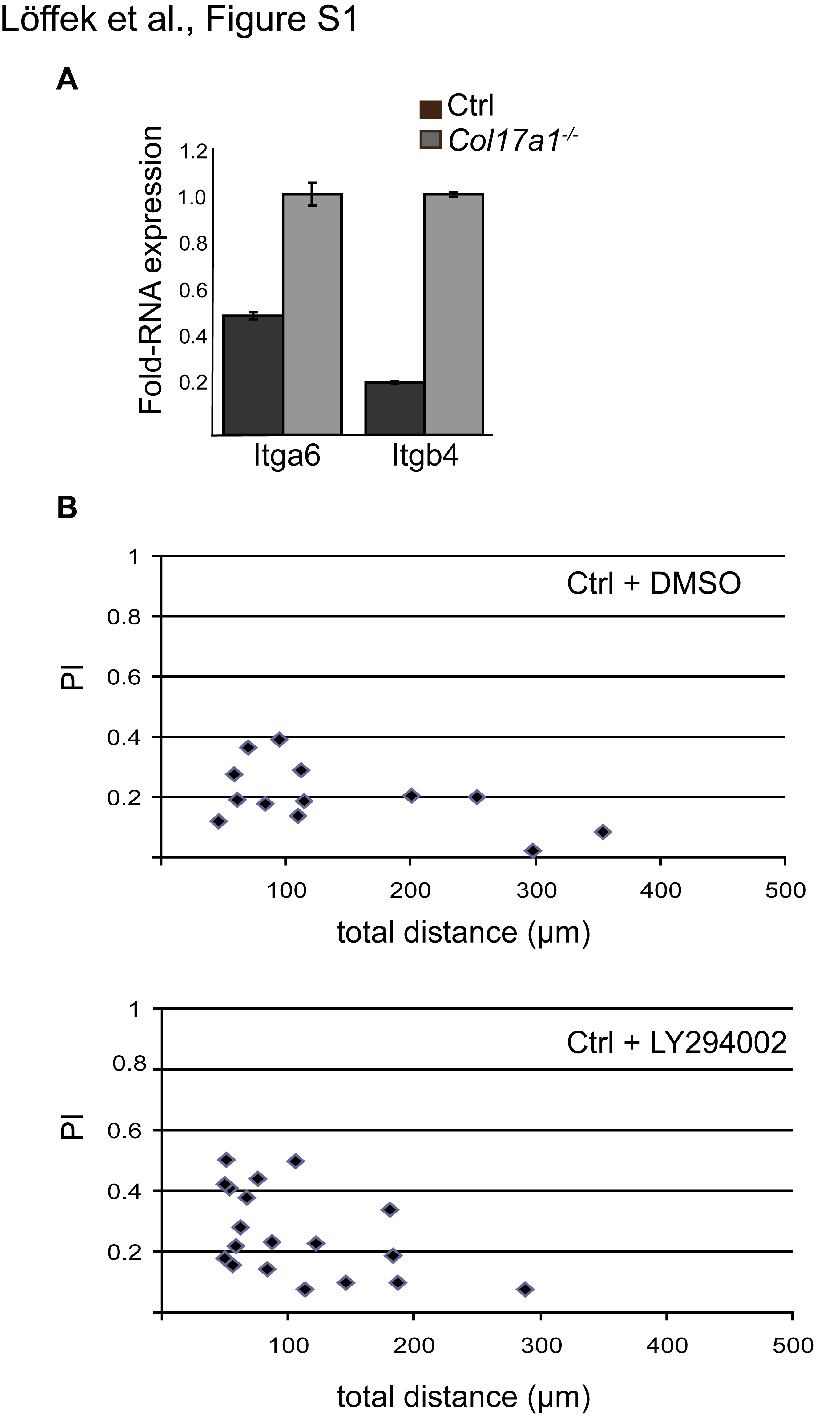

Supplement: Figure S1 — A, Quantitative RT-PCR of immortalized (high passage) control and Col17a1− /− keratinocytes (cells of four individuals per genotype have been analyzed; number of independent experiments = 3). B, Keratinocytes derived from wild type (Ctrl) mice were grown on glass-bottom culture dishes and treated with either DMSO or LY294002 [50 µM]. Cell migration was recorded by time-lapse imaging every 5 minutes during 4 hours. The distance migrated is indicated on the x-axis, the processive index (PI) on the y-axis. (TIF) [file pone.0087263.s001.tif]

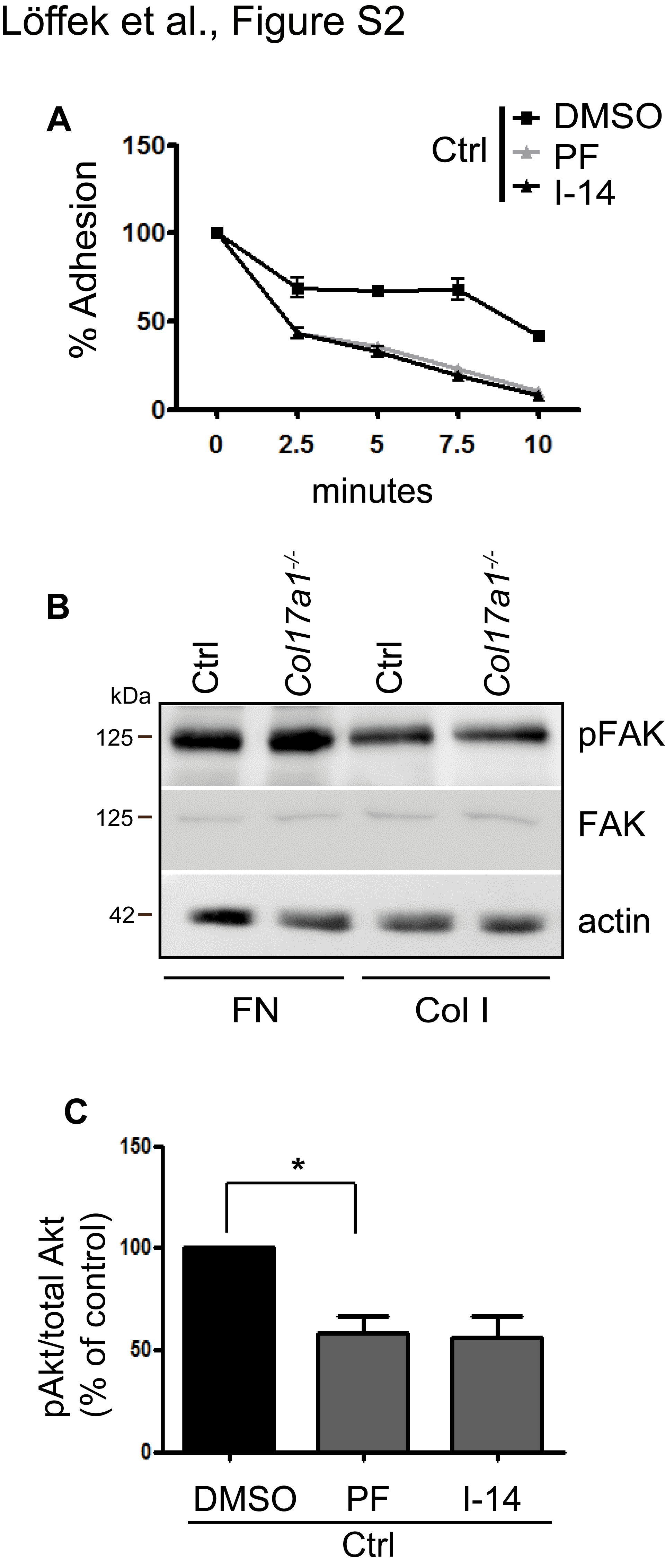

Supplement: Figure S2 — A, Keratinocytes derived from wild type (Ctrl) mice were treated with DMSO or different phospho-FAK inhibitors (PF 573228 [5 µM] and Inhibitor 14 [1 µM]) for 6 hours and thereafter subjected to the trypsin/EDTA detachment assay. The data are shown as mean ± SEM (cells of three individuals have been analyzed; number of independent measurements = 3). B, Keratinocytes derived from wild type (Ctrl) and Col17a1− /− mice were allowed to adhere to fibronectin (FN) and collagen I (Col I) for 2 hours, lysed and immunoblotted with antibodies to phospho-FAK (Y397), total FAK and actin. C, Keratinocytes isolated from wild type mice (Ctrl) were treated with DMSO or different phospho-FAK inhibitors (PF 573228 [5 µM] and Inhibitor 14 [1 µM]) for 6 hours and thereafter subjected to trypsin/EDTA detachment assay. The data are shown as mean ± SEM (cells of three individuals have been analysed, number of independent measurements = 3); *p<0.05. (TIF) [file pone.0087263.s002.tif]
